# Design of Peptide Substrate for Sensitively and Specifically Detecting Two Aβ-Degrading Enzymes: Neprilysin and Angiotensin-Converting Enzyme

**DOI:** 10.1371/journal.pone.0153360

**Published:** 2016-04-20

**Authors:** Po-Ting Chen, Chao-Long Chen, Lilian Tsai-Wei Lin, Chun-Hsien Lo, Chaur-Jong Hu, Rita P.-Y. Chen, Steven S.-S. Wang

**Affiliations:** 1 Institute of Biochemical Sciences, National Taiwan University, Taipei, 10617, Taiwan; 2 Institute of Biological Chemistry, Academia Sinica, Taipei, 11529, Taiwan; 3 Department of Chemical Engineering, National Taiwan University, Taipei, 10617, Taiwan; 4 Department of Neurology, Shuang-Ho Hospital, Taipei Medical University, Taipei, 110, Taiwan; Western University of Health Sciences, UNITED STATES

## Abstract

Upregulation of neprilysin (NEP) to reduce Aβ accumulation in the brain is a promising strategy for the prevention of Alzheimer’s disease (AD). This report describes the design and synthesis of a quenched fluorogenic peptide substrate qf-Aβ(12–16)AAC (with the sequence VHHQKAAC), which has a fluorophore, Alexa-350, linked to the side-chain of its C-terminal cysteine and a quencher, Dabcyl, linked to its N-terminus. This peptide emitted strong fluorescence upon cleavage. Our results showed that qf-Aβ(12–16)AAC is more sensitive to NEP than the previously reported peptide substrates, so that concentrations of NEP as low as 0.03 nM could be detected at peptide concentration of 2 μM. Moreover, qf-Aβ(12–16)AAC had superior enzymatic specificity for both NEP and angiotensin-converting enzyme (ACE), but was inert with other Aβ-degrading enzymes. This peptide, used in conjunction with a previously reported peptide substrate qf-Aβ(1–7)C [which is sensitive to NEP and insulin-degrading enzyme (IDE)], could be used for high-throughput screening of compounds that only upregulate NEP. The experimental results of cell-based activity assays using both qf-Aβ(1–7)C and qf-Aβ(12–16)AAC as the substrates confirm that somatostatin treatment most likely upregulates IDE, but not NEP, in neuroblastoma cells.

## Introduction

Alzheimer’s disease (AD) is one of the leading neurodegenerative diseases and the foremost cause of dementia. Other major death-causing diseases, such as cancer and cardiovascular diseases, are gradually declining due to the progress of medical research, but as our population ages, the number of people affected by AD is increasing with the increase in human life expectancy. The neuropathological hallmarks of AD include extracellular amyloid plaques, intracellular neurofibrillary tangles formed from phosphorylated tau, cerebrovascular amyloid deposits, and neuronal loss. The battle against AD has therefore involved considerable effort aimed at developing therapeutic strategies that focus on amyloid formation and tau phosphorylation as two major targets [1–4]. However, at present, only memantine and four cholinesterase inhibitors have been approved for symptomatic amelioration in patients with AD [2, 5].

The amyloid cascade hypothesis of AD holds that the excessive accumulation and abnormal aggregation of a notorious peptide, “amyloid β-protein” (Aβ; containing 39–43 amino acids), is linked to the onset of the neurodegenerative process. Aβis a catabolic product derived from a membrane protein, amyloid precursor protein (APP), formed during the cooperative cleavage by two proteolytic enzymes, β-secretase and γ-secretase. Mutations of APP and presenilin (a catalytic component of γ-secretase) lead to early onset of AD, thereby supporting accumulation of Aβas a key event in the early stage of AD progression. Application of anti-Aβ antibodies fails to ameliorate the cognitive and functional decline of AD patients but appears to be effective in Aβ clearance. This finding has recently changed the direction of clinical trials from AD therapy to AD prevention [6].

*In vivo*, Aβ is degraded by several endogenous Aβ-degrading enzymes, including neprilysin (NEP), plasmin, several matrix metalloproteases, insulin-degrading enzyme (IDE), endothelin-converting enzyme (ECE), and angiotensin-converting enzyme (ACE) [7]. Among these, NEP has the best correlation with Aβ accumulation. A deficiency of endogenous NEP increases the level of Aβ in the brains of NEP-knockout mice in a gene dose-dependent manner [8]. In a mouse study, the hippocampus, which is considered the most vulnerable brain region in AD pathology, had lower NEP and IDE expression levels when compared to the unaffected cerebellum. The expression of IDE and NEP in the hippocampus also decreases with increasing age [9]. A western blot analysis of human brains revealed a lower NEP expression level in AD samples than in normal samples [10] and the reverse-transcription polymerase chain reaction also revealed a reduction in NEP mRNA levels in AD brains [11]. Upregulation of NEP therefore may represent a possible preventive approach for treatment of AD. Increasing the NEP level in AD-transgenic mouse models, either by direct injection of NEP into the brain [12] or by transgenic or viral-mediated overexpression of NEP in the brain or peripheral tissues, reduced the Aβ load and improved memory impairment [13–17].

The screening of compounds capable of upregulating NEP requires an NEP-sensitive assay system. We previously designed a peptide, qf-Aβ(1–7)C, for detection of NEP activity [18]. This peptide has a fluorophore Alexa-350 linked to its C-terminal Cys side-chain and a quencher (Dabcyl) at its N-terminus. The fluorescence emission of Alexa-350 is quenched by the presence of Dabcyl nearby and strong fluorescence emission at 436 nm is only detected upon excitation at 346 nm when the peptide is cleaved by endopeptidases. This peptide is able to detect NEP at levels as low as 0.1 nM (about 1.6 ng) and only 2 μM of this peptide is required in the assay. The peptide substrate qf-Aβ(1–7)C is sensitive to both NEP and IDE and inert to other Aβ-degrading enzymes, such as ECE-1, ACE, plasmin, MMP-9, and MMP-3. In the present study, our initial aim was to employ the same design strategy to synthesize a peptide sensitive to other Aβ-degrading enzymes, but not to NEP, which would then serve as the control in compound screening. The peptide qf-Aβ(12–18)C (sequence VHHQKLVC) was cleaved by multiple Aβ-degrading enzymes, including NEP, ACE, and ECE-1, whereas the redesigned peptide qf-Aβ(12–16)AAC (sequence VHHQKAAC) was sensitive to NEP and ACE only. Moreover, qf-Aβ(12–16)AAC could be utilized to detect both NEP and ACE at levels as low as 0.03 nM. Notably, these synthesized peptide substrates had a superior sensitivity for NEP detection when compared to the previously reported peptide qf-Aβ(1–7)C (0.1 nM).

## Materials and Methods

### Synthesis of the quenched fluorogenic peptide substrates

Two peptides, Aβ(12–18)C (sequence VHHQKLVC, which corresponds to residues 12 to 18 of the Aβ peptide followed by a cysteine residue) and Aβ(12–16)AAC (sequence VHHQKAAC, which corresponds to residues 12 to 16 of the Aβ peptide followed by two alanine residues and one cysteine residue), were prepared by the Fmoc-polyamide method on a PS3 peptide synthesizer (Rainin, USA) [19]. The thiol-reactive Alexa-350 (Alexa Fluor^®^ 350 C_5_-maleimide) and amine-reactive Dabcyl [4-(4’-N, N-dimethylaminophenyl)azobenzoic acid, succinimidyl ester] (Invitrogen) were used as the fluorescence donor and quencher, respectively. About 1 mg of peptide was dissolved in 10 mM Tris-HCl/DMSO [1/2.3 (v/v), pH 7.2]. Tris (2-carboxyethyl) phosphine (TCEP) was dissolved in DMSO to make a 50 mM stock solution. Alexa-350 was dissolved in DMSO to make a 20 mM stock solution. The peptide solution was mixed with one half of an equivalent volume of TCEP and kept on ice. Alexa-350 was added dropwise to a final molar ratio of peptide:Alexa-350 = 1:4. The mixture was reacted overnight in the dark at room temperature with gentle inversion (91 rpm). The dye-labeled peptide was purified by HPLC and identified on a MALDI mass spectrometer (MALDI micro MX, Waters, USA). The quenching group was added by dissolving Alexa-350-labeled peptide and Dabcyl (molar ratio peptide: Dabcyl = 1:10) in DMSO. A 1/20 volume of 100 mM sodium bicarbonate was slowly added and the mixture was reacted with gentle inversion for 2 hr at room temperature in dark. The double-labeled peptides, designated qf-Aβ(12–18)C and qf-Aβ(12–16)AAC, were purified by HPLC and identified on a MALDI mass spectrometer (MALDI micro MX, Waters, USA). The resulting purified peptides were lyophilized and stored at -20°C.

### Fluorescence spectroscopy

Peptides were dissolved in DMSO to make a 1 mM stock solution and then 2 μM qf-Aβ(12–18)C or qf-Aβ(12–16)AAC was reacted at 37°C for 1 hr with 2 nM of the indicated enzymes in the buffers suggested by the manufacturer (see below). The fluorescence emission spectrum between 370 and 600 nm was recorded in a 3-mm path-length rectangular cuvette on a FP-750 spectrofluorometer (Jasco, Japan) with excitation at 346 nm.

### Aβ-degrading enzymes

Recombinant human NEP (0.5 mg/mL), insulin-degrading enzyme (IDE) (0.386 mg/mL), endothelin-converting enzyme 1 (ECE-1) (0.298 mg/mL), matrix metalloproteinases [(MMP)-2 (0.373 mg/mL), MMP-3 (0.124 mg/mL), and MMP-9 (0.5 mg/mL)], and angiotensin-converting enzyme (ACE) (0.434 mg/mL) were purchased from R&D Systems (USA). Human plasmin (1 mM) was purchased from Sigma (USA). All the enzymes purchased were marked with purity >90% (The characterization of the purity of some of the enzymes used can be seen in S1 Fig). The enzymes were diluted in the buffers as suggested by the manufacturer: 50 mM Tris-HCl (pH 7.5), 25 mM NaCl, 5 μM ZnCl_2_ for NEP, IDE, and ACE; 0.1 M MES, 0.1 M NaCl (pH 6) for ECE-1; 50 mM Tris-HCl (pH 7), 150 mM NaCl, 10 mM CaCl_2_, 0.05% Brij-35 for MMP-2, MMP-3 and MMP-9; and 50 mM Tris-HCl (pH 7.5) for plasmin. A pre-activation step was required for MMP-2, MMP-3, and MMP-9. A 2 mM stock of solution of p-aminophenylmercuric acetate was prepared in DMSO and then mixed with a 1:1 volume of MMP-2, MMP-3, or MMP-9. The reaction mixture was incubated with shaking at 37°C for 24 hr.

### Sensitivity of qf-Aβ(12–18)C and qf-Aβ(12–16)AAC detection of various Aβ-degrading enzymes

Different concentrations of enzymes were reacted with 2 μM qf-Aβ(12–18)C or qf-Aβ(12–16)AAC on a 96-well plate in the dark, without or with incubation for 1, 2, and 3 hr at 37°C. The fluorescence intensities were then measured with excitation at 360 nm and emission at 465 nm on a Paradigm^™^ Detection Platform (Beckman Coulter, USA). The data shown are the averages of three independent samples.

### Somatostatin treatment of human neuroblastoma SH-SY5Y cells

Human SH-SY5Y cells (ATCC, USA) were cultured in Dulbecco’s modified Eagle’s medium (Gibco, USA) supplemented with 10% fetal bovine serum (FBS; Biological Industries, USA) in 5% CO_2_ at 37°C. The cells were harvested, suspended at a density of 5 × 10^5^ cells/mL in the same medium without FBS, and 1 mL was plated in each well of a 12-well plate. Somatostatin (Sigma, USA) was added to the cultures at a final concentration of 1 μM and incubated for 24 hr. After incubation, the culture medium was replaced with fresh medium, and then 2 μM qf-Aβ(1–7)C and qf-Aβ (12–16)AAC were added individually to each well. After a 1 hr incubation, 100 μL of medium was removed for fluorescence measurement on a Paradigm^™^ Detection Platform (Beckman Coulter, USA) with excitation at 360 nm and emission at 465 nm. The data shown are the averages of at least three independent samples.

### Statistical analysis

All data are expressed as average ± standard deviation (S.D.) for three independent determinations. The significance of the results was determined with Student’s t-test. Unless otherwise noted, a criterion of p < 0.01 was employed to determine whether the sample set and the untreated control set were statistically different. Asterisks above indicate the significance of the result relative to the untreated control: a single asterisk indicates a value of p < 0.01, double asterisks indicate a value of p < 0.001, and triple asterisks indicate a value of p < 0.0001 (*: p < 0.01; **: p < 0.001; ***: p < 0.0001).

## Results and Discussion

As pointed out in many research papers, short peptides synthesized with double fluorescent dye labels are useful tools for detecting the activity of endopeptidases; however, other studies have also suggested that these double-labeled substrates are difficult to synthesize and are normally recovered in low yields. The present study adopted chemical synthesis using Fmoc chemistry for first synthesizing the backbones of the peptide substrates (e.g., Aβ(12–18)C and Aβ(12–16)AAC). The subsequent addition of two different fluorescent dyes—a fluorescence reporter/donor Alexa-350 and a fluorescence quencher Dabcyl—to these backbones yielded the double-labeled peptide substrates, qf-Aβ(12–18)C and qf-Aβ(12–16)AAC, which were then used to evaluate endopeptidase enzyme activity. Alexa-350 and Dabcyl were attached to the residues at the C-terminus and the N-terminus, respectively. Alexa-350, contains the functional group, maleimide, which can react with thiol groups on proteins. Maleimide can form a stable covalent bond quickly with cysteine side chains on proteins in a neutral environment. Dabcyl can also attach sulfhydryl groups, so its attachment to both termini was prevented by adding Alexa-350 first, to react with the side chain of the C-terminal cysteine. The Alexa-350-carrying peptides were then purified and further reacted to incorporate Dabcyl at the N-terminus.

### Specificity and sensitivity of qf-Aβ(12–18)C for detecting various Aβ-degrading enzymes

**Fig 1:**
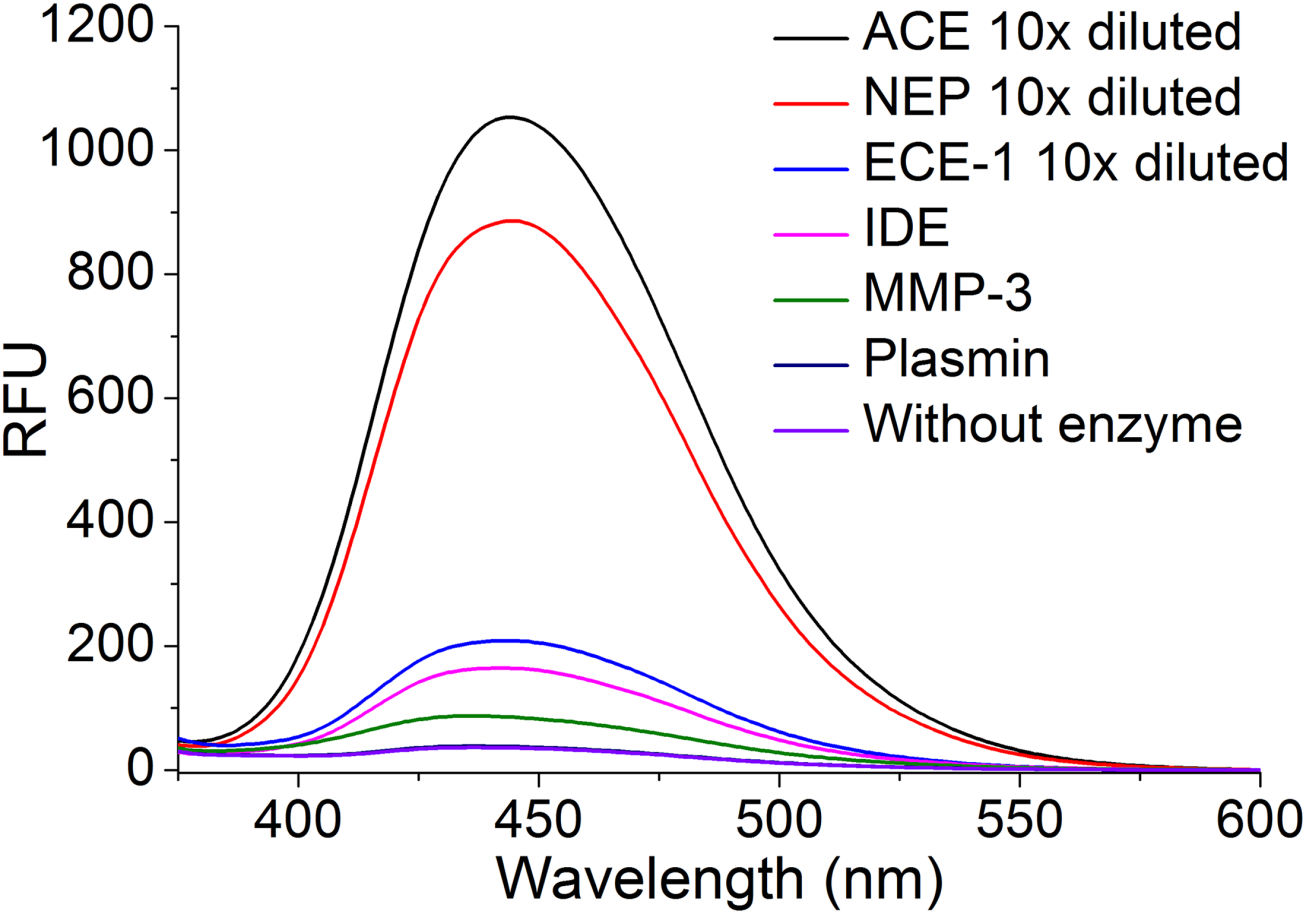
Fluorescence emission spectra of qf-Aβ(12–18)C treated with NEP, ACE, ECE-1, IDE, MMP-3, and plasmin. qf-Aβ(12–18)C (2 μM) was reacted with the indicated enzymes (2 nM) at 37°C for 1 hr. The samples digested by NEP, ACE, and ECE-1 were diluted 10 times before the fluorescence measurement due to the strong fluorescence intensity. The excitation wavelength was 346 nm.

**Fig 2:**
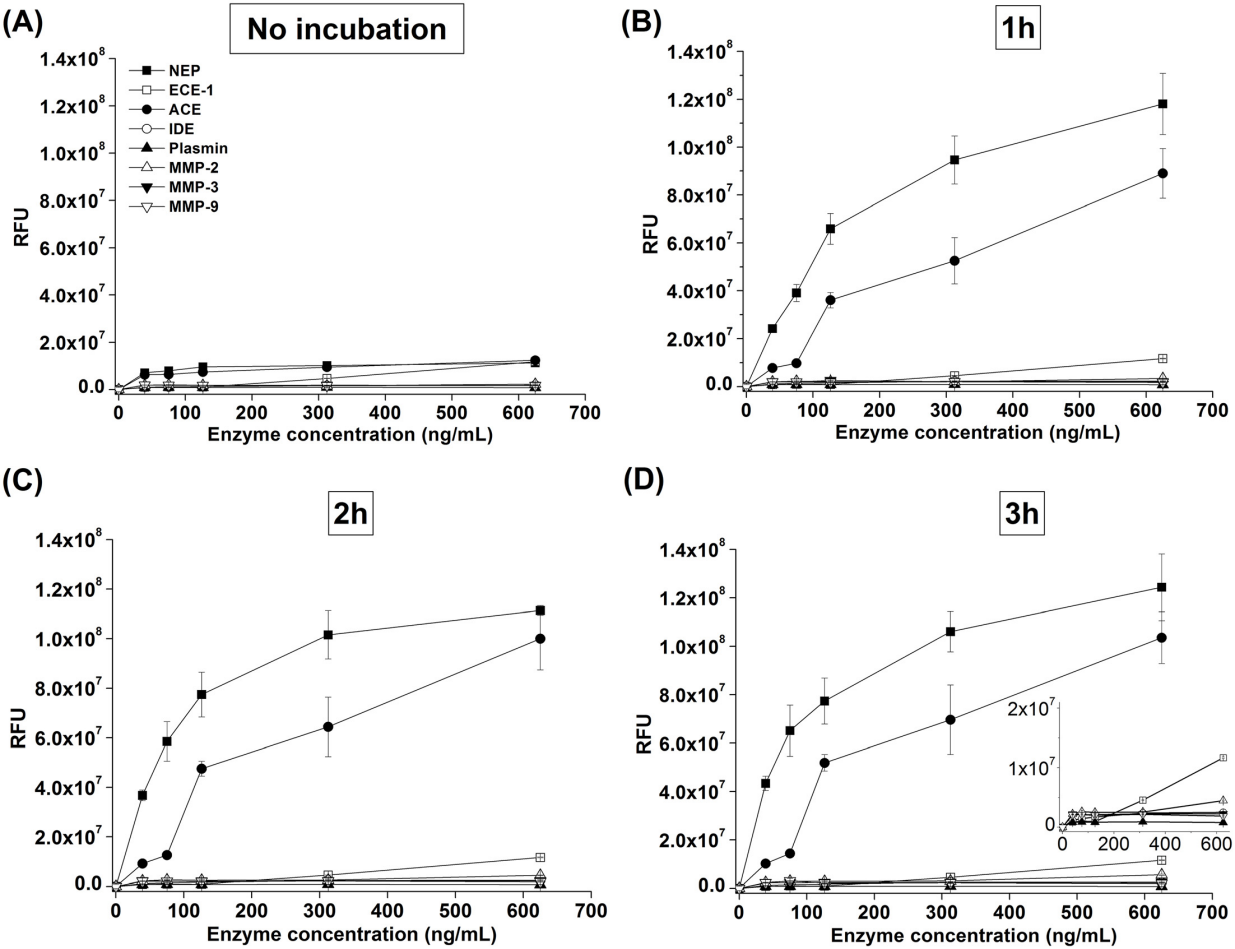
The specificity of qf-Aβ(12–18)C for various enzymes. qf-Aβ(12–18)C (2 μM) was reacted with different concentrations of the indicated enzymes without or with incubation at 37°C for 1, 2, and 3 hr. Inset in (D): the extension of the y-axis. The fluorescence intensity was measured at 360_Ex_/465_Em_.

We chose the Aβ sequence 12–18 for our designed peptide because it contains several reported cutting sites for IDE, ECE-1, and MMPs [7, 20–22]. We evaluated the sensitivity of our synthesized qf-Aβ(12–18)C for various Aβ-degrading enzymes by reacting 2 μM peptide with 2 nM of different enzymes at 37°C for 1 hr and recording the fluorescence spectra (Fig 1). The fluorescence emission at ~450 nm showed a very strong and unexpected fluorescence intensity following peptide treatment with NEP and ACE. Other enzymes, at higher concentrations, were also tested for cleavage of qf-Aβ(12–18)C by monitoring the fluorescence emission of a mixture of 2 μM qf-Aβ(12–18)C and a range of concentrations (39, 75, 162, 312, or 625 ng/mL) of NEP, ACE, ECE-1, IDE, MMP-2, MMP-3, MMP-9, and plasmin, without or with incubation at 37°C for 1, 2, and 3 hr (Fig 2). NEP and ACE showed very strong cleavage activity, whereas and ECE-1 showed mild activity. Plasmin, IDE, and the three MMPs showed no significant fluorescence increase even at the highest enzyme concentration of 625 ng/mL (equal to ~ 6 nM IDE).

**Fig 3:**
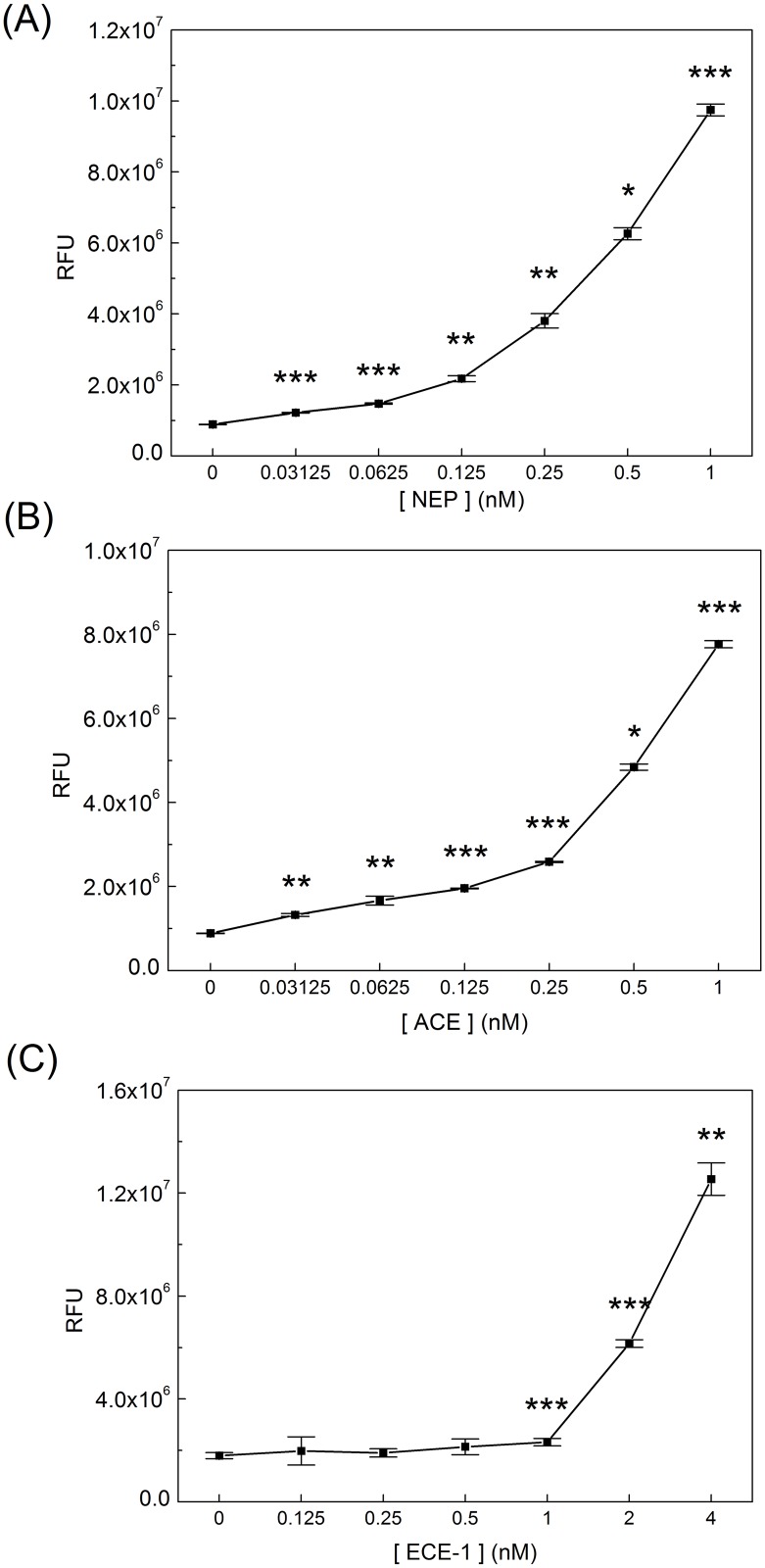
The sensitivity of qf-Aβ(12–18)C for various enzymes. qf-Aβ(12–18)C (2 μM) was reacted with different concentrations of the indicated enzymes at 37°Cfor 1 hr. The fluorescence measurements were performed at the excitation and emission wavelengths of 360 and 465 nm, respectively (*: p<0.01; **: p<0.001; ***: p<0.0001).

The purpose of this study was to design a peptide substrate that could be used for a rapid and sensitive detection of Aβ-degrading enzymes. The detection of NEP, ACE, and ECE-1 at sub-nanomolar concentrations by qf-Aβ(12–18)C was examined using a 1-hr reaction time. As shown in Fig 3, qf-Aβ(12–18)C could detect NEP and ACE at concentrations as low as 0.03 nM. The sensitivity of qf-Aβ(12–18)C for ECE-1 was much lower, with no detection observed at ECE-1 concentrations lower than 1 nM.

### Specificity and sensitivity of qf-Aβ(12–16)AAC for detecting various Aβ-degrading enzymes

The observation that qf-Aβ(12–18)C peptide showed a high sensitivity for NEP and ACE, but a relatively low sensitivity for ECE-1 suggested that its enzyme specificity could be improved by decreasing its susceptibility to ECE-1 activity. The location of the ECE-1 cutting site in the Aβ peptide was reported as the N-terminal Leu17 and Val18 [21]; therefore, we redesigned the sequence of the peptide qf-Aβ(12–18)C by replacing Leu17 and Val18 with two Ala residues to yield the peptide substrate designated qf-Aβ(12–16)AAC.

**Fig 4:**
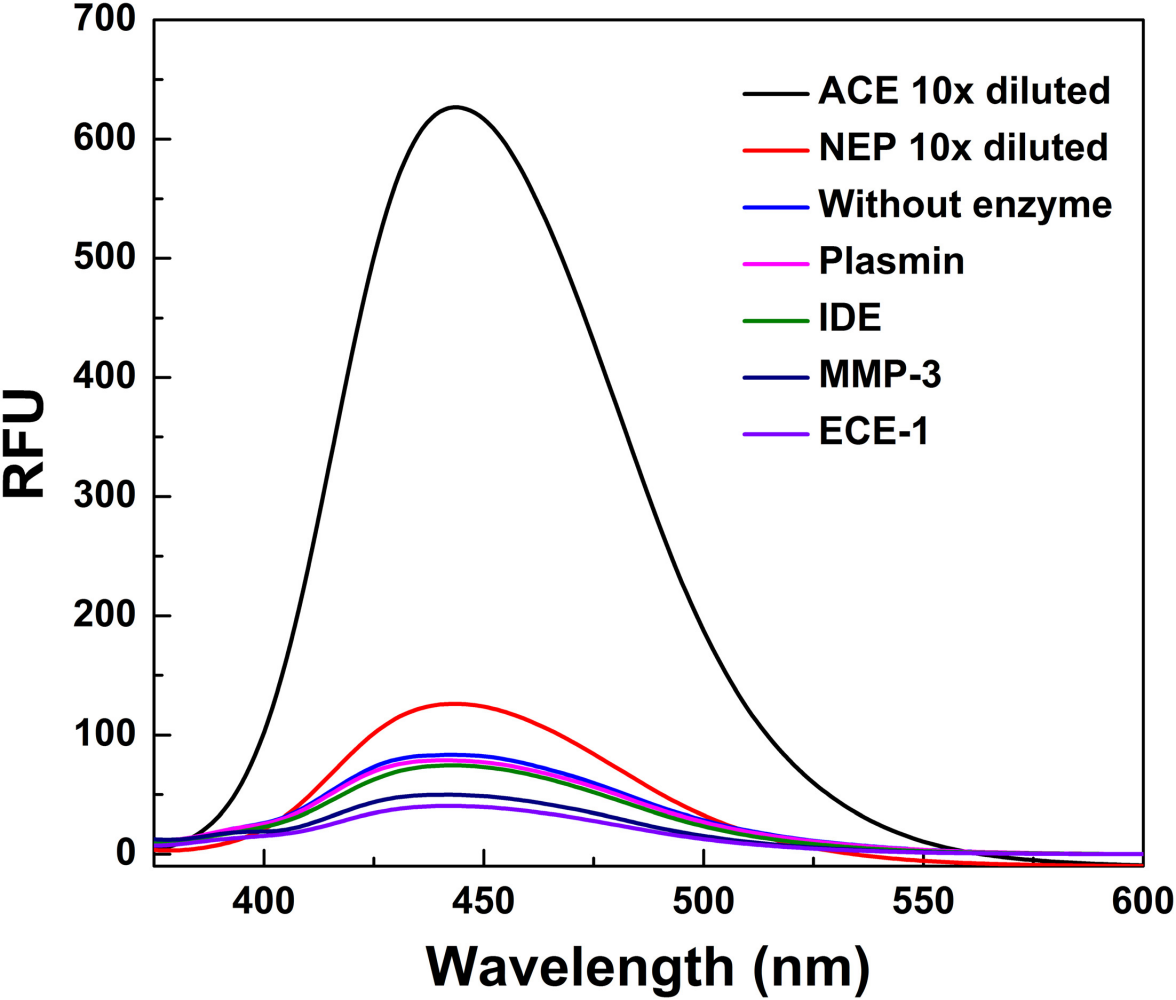
Fluorescence emission spectra of qf-Aβ(12–16)AAC treated with NEP, ACE, ECE-1, IDE, MMP-3, and plasmin. qf-Aβ(12–16)AAC (2 μM) was reacted with the indicated enzymes (2 nM) at 37°C for 1 hr. The samples digested by NEP and ACE were diluted 10 times before the measurement due to strong fluorescence intensity. The excitation wavelength used in the fluorescence measurements was 346 nm.

**Fig 5:**
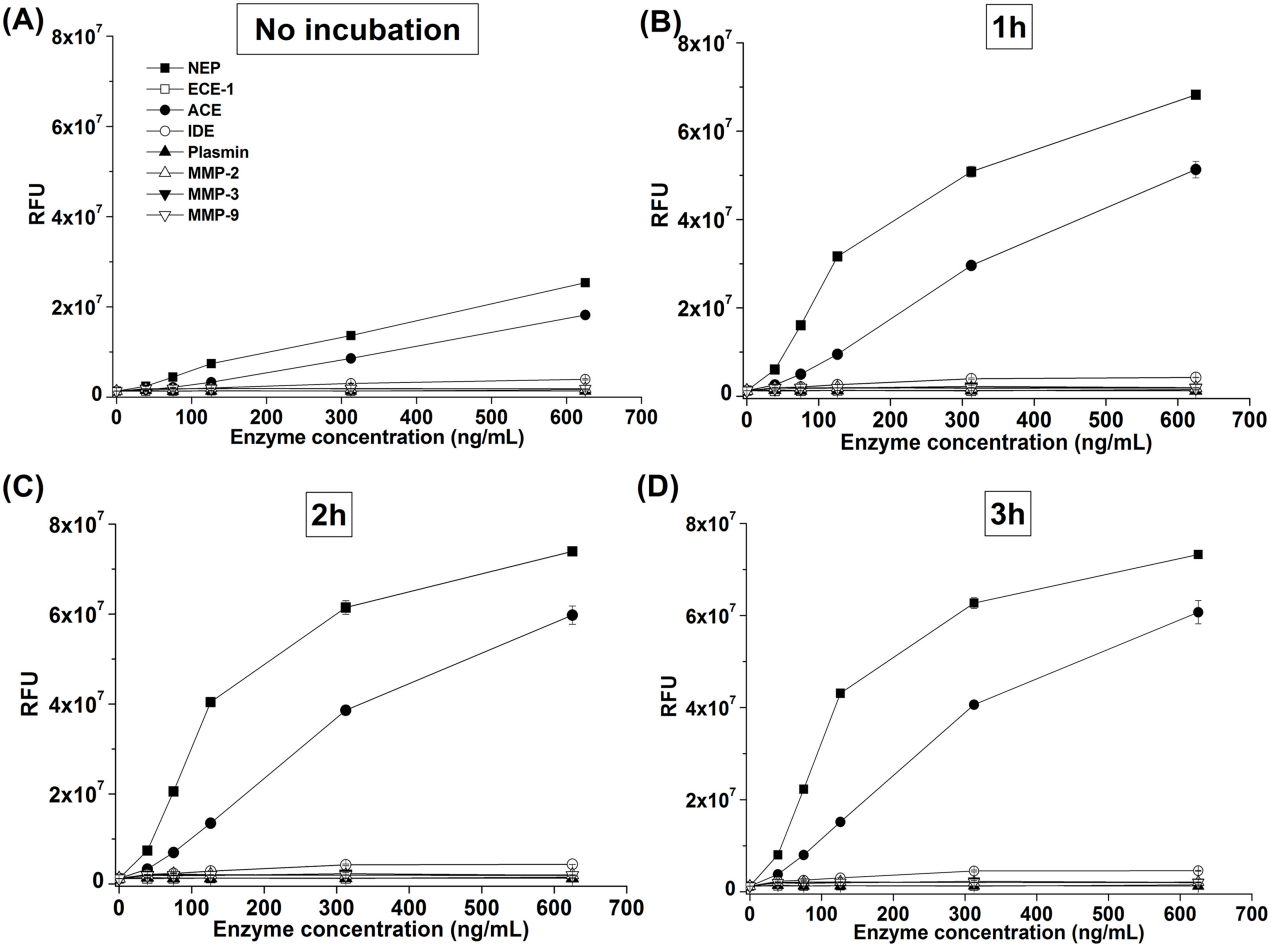
The specificity of qf-Aβ(12–16)AAC for various enzymes. qf-Aβ(12–16)AAC (2 μM) was reacted with different concentrations of the indicated enzymes without or with incubation at 37°C for 1, 2, and 3 hr. Inset in (D): the extension of on the y-axis. The fluorescence measurements were performed at the excitation and emission wavelengths of 360 and 465 nm, respectively.

**Fig 6:**
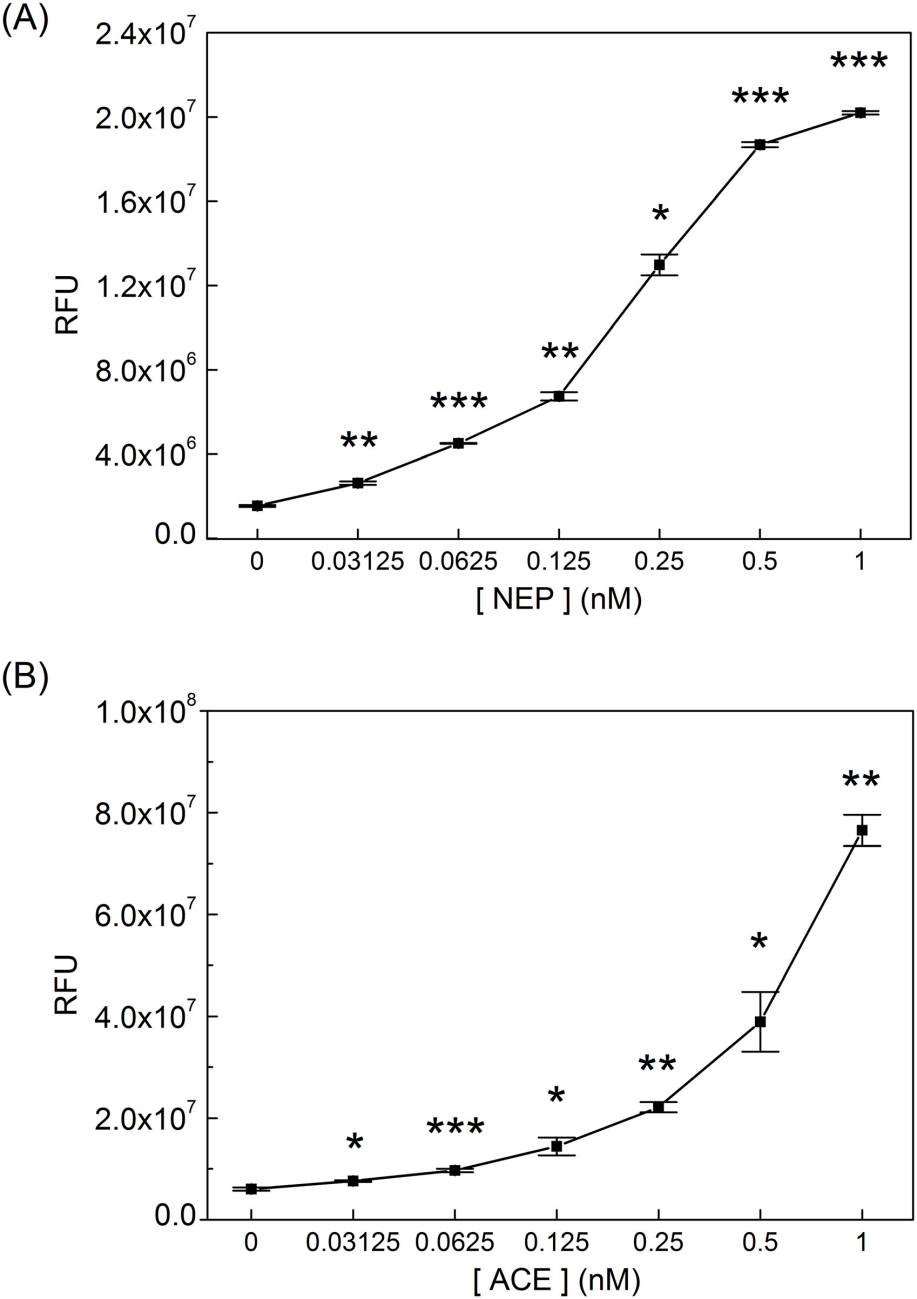
The sensitivity of qf-Aβ(12–16)AAC for various enzymes. qf-Aβ(12–16)AAC (2 μM) was reacted with different concentrations of the indicated enzymes at 37°Cfor 1 hr. The fluorescence measurements were performed at the excitation and emission wavelengths of 360 and 465 nm, respectively (*: p<0.01; **: p<0.001; ***: p<0.0001).

We evaluated the sensitivity of qf-Aβ(12–16)AAC in the same way as qf-Aβ(12–18)C. Comparison of the data depicted in Fig 4 with those in Fig 1 confirms that NEP and ACE could digest both peptides, whereas ECE-1 could digest qf-Aβ(12–18)C but not qf-Aβ(12–16)AAC. The fluorescence intensities of qf-Aβ(12–16)AAC treated with different concentrations of various Aβ-degrading enzymes are shown in Fig 5. ECE-1 could not digest qf-Aβ(12–16)AAC, even at high enzyme concentrations (e.g., 625 ng/mL, which is equivalent to ~7.9 nM ECE-1). The sensitivity of qf-Aβ(12–16)AAC to NEP and ACE was similar to that of qf-Aβ(12–18)C (Fig 6). The values of kinetic parameters (Km and kcat) for digestion of the substrate (qf-Aβ(12–16)AAC) by the enzymes (e.g., NEP and ACE) have also been determined by conducting the enzyme-substrate reaction experiments, followed by fitting the Michaelis-Menten model equation to the results of initial rate/velocity *v*.*s*. substrate (qf-Aβ(12–16)AAC) concentration, which were obtained from the kinetic data of the aforesaid experiments, using the non-linear regression. Evidently, the values of Km and kcat were not found to be statistically different between the NEP-substrate system and the ACE-substrate system (see S2 Fig and S1 Table).

To further confirm (a) if the discrete single sites were cleaved and (b) if the hydrolysis occurred at the same bond as that of the physiological Aβ peptide. We have performed mass spectrometric analyses on the purified peptide fragments of the substrate qf-Aβ(12–16)AAC upon hydrolysis/cleavage by NEP or ACE using MALDI-TOF mass spectrometer. As demonstrated in Fig (A)-(B) in S1 File and S2 Table, the observed mass [M+H^+^] of the peptide fragment obtained from NEP digestion was determined to be 741.187 Da (see (B)), which matches the theoretical mass of the peptide fragment AAC-Alexa-350 (740 Da). This result indicated that the cutting site of NEP is between residues K and A in the sequence “VHHQKAAC”. As for the case of ACE digestion (see Fig (C)-(D) in S1 File and S2 Table), the observed mass [M+H^+^] in (D) was determined to be 670.130 Da, which is ~71 Da lower than the value obtained in the case of NEP digestion. Since this amount of mass reduction is equal to the mass of Ala residue, we believe the peak belongs to the peptide fragment AC-Alexa-350. This finding indicated that the cutting site of ACE is located between two Ala residues in the sequence “VHHQKAAC”.

#### Cell-based activity assay to screen compounds that could upregulate NEP

**Fig 7:**
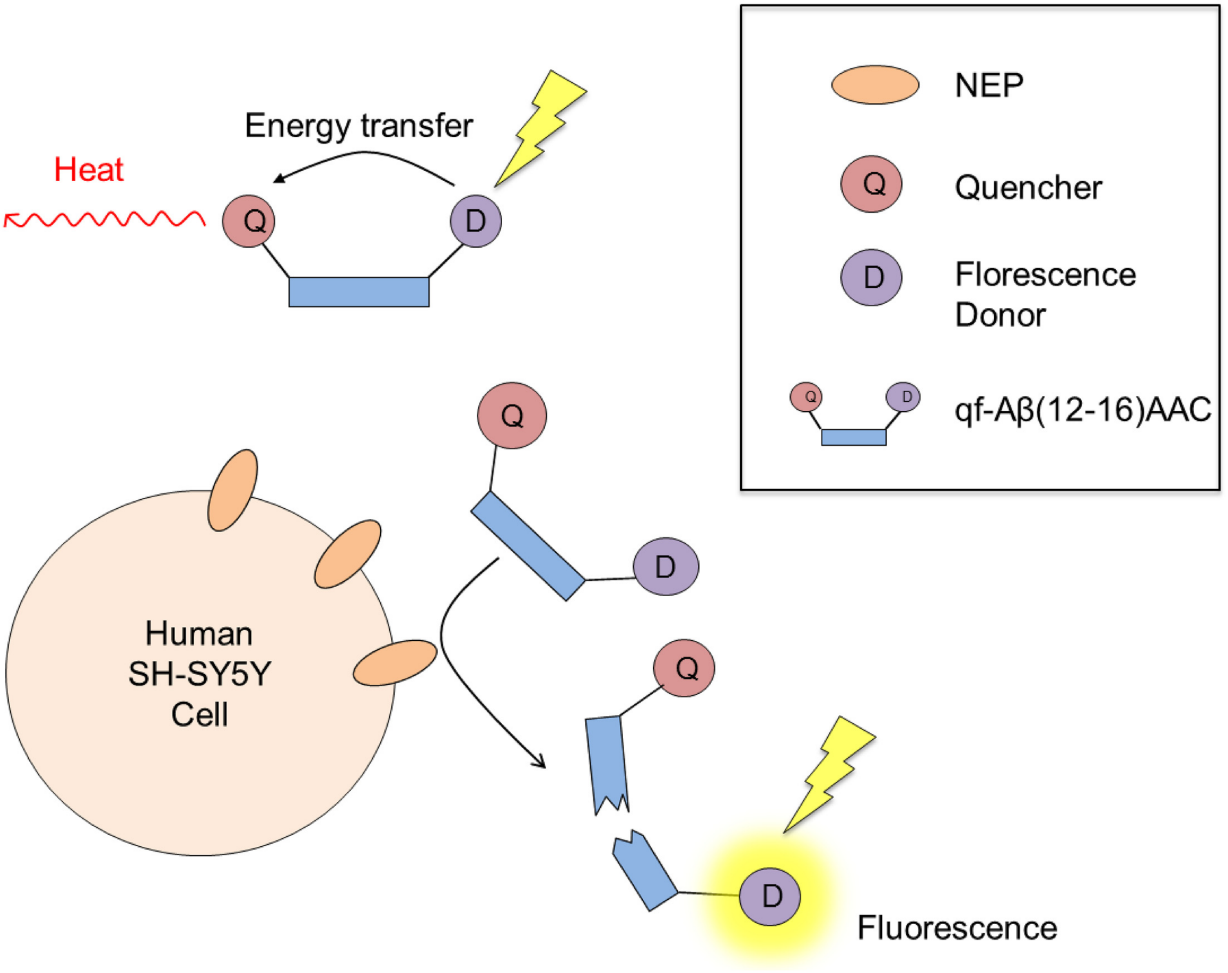
Illustration of the cell-based NEP activity assay using qf-Aβ(12–16)AAC as the peptide substrate. Human neuroblastoma SH-SY5Y cells were used in the cell-based NEP activity assay.

**Fig 8:**
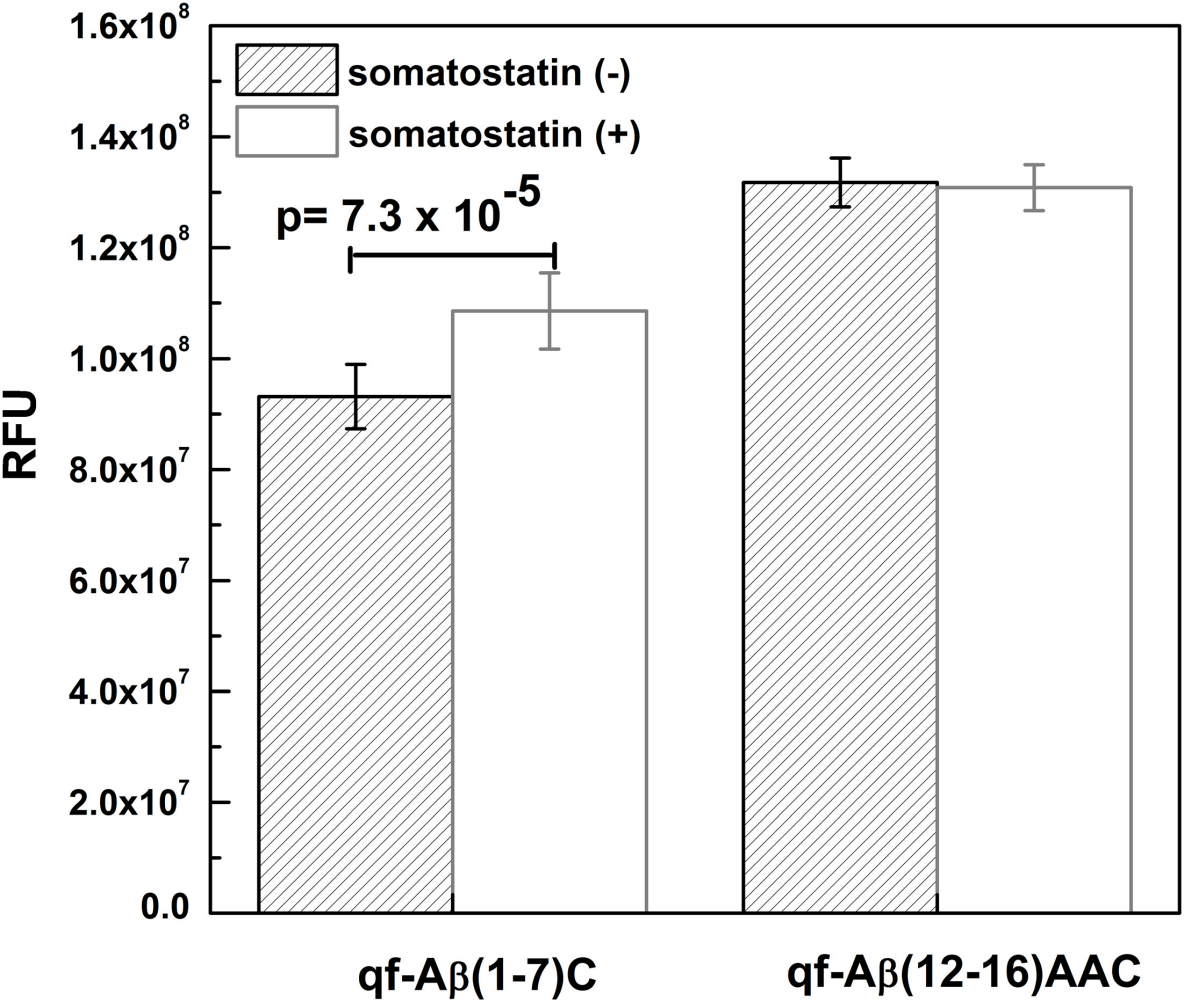
The effect of somatostatin treated SH-SY5Y cells on the digestion ability of the peptide substrate qf-Aβ(1–7)C or qf-Aβ(12–16)AAC present in the culture medium.

NEP is one of the major Aβ-degrading enzymes and upregulation of NEP is a promising strategy for increasing Aβ clearance to prevent the development of AD. Searching for chemicals with the ability to upregulate NEP activity is a more feasible approach for AD prevention, when compared with viral infection or stem cell transplantation. Our results showed that qf-Aβ(12–16)AAC could be used to screen compounds that upregulate NEP or ACE in human neuroblastoma SH-SY5Y cells (Fig 7). In addition, the previously reported peptide substrate qf-Aβ(1–7)C is sensitive to NEP and IDE but inert to other Aβ-degrading enzymes [18]. Therefore, a combination of these two peptide substrates could be used to cross-examine screened compounds that can activate NEP only. For example, Saito et al. used glutaryl-Ala-Ala-Phe-methoxy-2-naphthylamide as the peptide substrate in an enzyme activity assay and proposed that somatostatin is capable of regulating NEP activity in the primary cortical neurons [23]. On the contrary, Tundo et al. reported that somatostatin regulated IDE activity by affecting its expression and secretion in microglia cells, as revealed by western blotting and ELISA [24]. The addition of qf-Aβ(1–7)C and qf-Aβ(12–16)AAC individually to somatostatin-treated cell cultures resulted in enhanced fluorescence intensity only in the culture containing added qf-Aβ(1–7)C, but not qf-Aβ(12–16)AAC (Fig 8). This finding from cell-based screening analysis leads to the apparent conclusion that somatostatin is able to upregulate IDE, but not NEP.

To further verify this conclusion obtained from the cell-based assay system, we have also performed the western blotting analysis to examine the influence of somatostatin on the level of membrane-associated IDE in both the undifferentiated and retinoic acid-differentiated SH-SY5Y cells. Our results evidently showed that a slight increase in the amount of membrane-associated IDE was observed in both the undifferentiated and retinoic acid-differentiated SH-SY5Y cells upon somatostatin treatment (shown in S3 Fig). The result that IDE expression is elevated in the presence of somatostatin is consistent with the finding in our previous study that a slight increase in IDE activity was detected in the assay [18].

### Comparison with other peptide substrates

The expression of NEP is low in neuronal cells; therefore, the development of a very sensitive detection system is critical when attempting to compare the levels of NEP activities among the samples containing neuronal cells. Several peptide substrates have been reported for assaying NEP activity. For instance, Saito et al. [22] used a concentration of glutaryl-Ala-Ala-Phe-methoxy-2-naphthylamide (Sigma) of 500 μM and this peptide substrate could be digested by many other proteases [23]. SAAP-AMC (Suc-L-Ala-L-Ala-L-Phe-7-amido-3-methylcoumarin, Sigma) [25, 26] was used to screen natural compounds that could increase NEP activity in the neuroblastoma SK-N-SH cells. The substrate concentration was 44 μM and a subsequent digestion step by aminopeptidase N was required. A similar response (i.e., detection of NEP and ACE) to that reported in the present study for qf-Aβ(12–16)AAC was observed for 3-dansyl-D-Ala-Gly-p-(nitro)-Phe-Gly (DAGNPG, also named dansyl-^D^AGF(*p*NO_2_)G, Sigma) [16]. However, a 40 μM DAGNPG concentration was required to detect 2 nM NEP [18]; therefore, the sensitivity of DAGNPG is clearly not as good as that of qf-Aβ(12–16)AAC.

## Conclusions

This work examined the possibility of using specifically designed peptide substrates for the detection of Aβ-degrading enzymes, which have been suggested as a potential targets in therapeutic strategies for AD. The synthesized peptide qf-Aβ(12–16)AAC exhibited excellent sensitivity and specificity as a substrate for NEP and ACE, highlighting its potential for seeking compounds/drugs that alter the levels of these Aβ-degrading enzymes. The combined use of the substrates qf-Aβ(1–7)C and qf-Aβ(12–16)AAC can also aid in high-throughput screening of compounds that specifically upregulate NEP alone. We also demonstrated that somatostatin is likely to increase the level of IDE but not NEP in neuroblastoma cells.

## Supporting Information

S1 Table Kinetic parameters for the hydrolysis of qf-Aβ(12–16)AAC by NEP and ACE. Digested peptide concentration was calculated according to the fluorescence intensity of qf-Aβ(12–16)AAC obtained from the reaction of NEP or ACE at 37°C overnight.
(DOC)

S2 Table Summary of the sequences, theoretical masses [M], and observed masses [M+H^+^] of the peptide fragments obtained from the MALDI spectrometric analysis shown in S1 File. Dabcyl-VHHQKAAC-Alexa350 is the parent peptide.
(DOC)

S1 Fig 8% Bis-Tris SDS-PAGE of NEP (102 kDa), ACE (160–180 kDa), and IDE (105 kDa). The numbers in the parentheses are the molecular masses of the enzymes obtained from the data sheets provided by the vendors.
(DOC)

S2 Fig Kinetics of qf-Aβ(12–16)AAC digestion by NEP and ACE. 2 nM NEP or ACE was reacted with 1 to 15 μM of qf-Aβ(12–16)AAC in 50 mM Tris-HCl (pH 7.5), 25 mM NaCl, 5 βM ZnCl_2_ at 37. The fluorescence emission at 465 nm was recorded every minute on a Paradigm^™^ Detection Platform (Beckman Coulter, USA) with excitation at 360 nm. The initial digestion rates for different concentrations of substrate were obtained by non-linear fitting and plotted against the substrate concentrations. The curve fitting on the data of initial rate/velocity versus substrate concentration plot was conducted according to the standard Michaelis-Menten equation.
(DOC)

S3 Fig Effect of somatostatin on IDE level by western blotting. SH-SY5Y cells were seeded in 100-mm dishes (10 mL; cell density of 7×10^5^ cells/mL), then, after 1 day, the medium was replaced with the fresh DMEM/F12 medium supplemented with 10% FBS and 10 μM of samatostatin (Sigma, USA) or vehicle. After incubation for 24 hr, cells were first detached from the plate with PBS and then centrifuged at 200 g at 4°C for 5 min. For the detection of IDE level in the differentiated cells, 5×10^5^ cells/mL in DMEM/F12 medium supplemented with 1% FBS was used. On the second day of incubation, the medium was replaced by the above-mentioned medium containing 10 μM retinoic acid (RA) and the cell culture was further incubated for 5 days (the medium should be changed every two days). The membrane fractions were extracted from the cell pellets using the Mem-PER Plus Membrane Protein Extraction Kit (Thermo Scientific, USA). After quantifying the protein content of the membrane fractions using a BCA kit (Thermo Scientific, USA), 50 μg of protein were first taken and resolved on an 8% Bis-Tris gels by SDS-PAGE and then transferred to a nitrocellulose membrane (PerkinElmer, USA). Next, the membrane was blocked with 5% non-fat dry milk (Fonterra, New Zealand) in the blocking buffer (Tris-buffered saline (TBS) containing 0.1% Tween 20) for 1 hr at 4°C, and incubated overnight at 4°C with mouse monoclonal anti-IDE antibody (Covance, USA) and mouse monoclonal anti-glyceraldehyde 3-phosphate dehydrogenase (GAPDH) antibody (Proteintech, USA) (1:1000 in the blocking buffer). After 3 washes with TBS, the membranes were incubated for 1 hr at 4°C with horseradish peroxidase-conjugated anti-mouse IgG antibodies (1:1000 in the blocking buffer, R&D Systems, USA). A slight increase in IDE amount was detected after somatostatin treatment.
(DOC)

S1 File Analysis of the cutting site of NEP or ACE on qf-Aβ(12–16)AAC. 1 mL 10 μM qf-Aβ(12–16)AAC in 50 mM Tris-HCl (pH 7.5), 25 mM NaCl, 5 μM ZnCl_2_ was reacted with 250 ng NEP or ACE at 37°C for 1 hr. The reaction product was purified by HPLC with C18 column. (A) HPLC chromatogram for NEP digestion. (C) HPLC chromatogram for ACE digestion. The elution gradient used was 0–50% Buffer B in 20 min. Buffer A: 5% acetonitrile/0.1% TFA in water; Buffer B: 0.1% TFA in acetonitrile. The products were detected by monitoring the absorbance at the wavelengths of 346 and 453 nm. After cleavage, the peptide fragment containing the Alexa-350 moiety exhibited a positive absorption peak at 346 nm but a negative absorption peak at 453 nm, which is due to the emitted fluorescence from Alexa-350. However, the peptide fragment containing the Dabcyl moiety showed absorption at 453 nm. The peak eluted at 12 min (with absorption at 346 nm) was collected and further identified/characterized by MALDI-TOF mass spectroscopy. (B) MALDI-TOF mass spectrum of the sample pointed with an arrow in the chromatogram of NEP digestion product. (D) MALDI-TOF mass spectrum of the sample pointed with an arrow in the chromatogram of ACE digestion product. The observed mass [M+H^+^] in (B) was determined to be 741.187 Da, which corresponds to the mass of the peptide fragment AAC-Alexa-350 with a theoretical mass of 740 Da. This result indicated that the cutting site of NEP is located between the residues K and A in the sequence “VHHQKAAC”. The observed mass [M+H^+^] in (D) was determined to be 670.130 Da, which is ~71 Da lower than the value obtained in the case of NEP digestion. Since this amount of mass reduction is equal to the mass of Ala residue, we believe the peak belongs to the peptide fragment AC-Alexa-350. This finding indicated that the cutting site of ACE is located between two Ala residues in the sequence “VHHQKAAC”.
(DOC)
