# Supplementary material for: Design of Peptide Substrate for Sensitively and Specifically Detecting Two Aβ-Degrading Enzymes: Neprilysin and Angiotensin-Converting Enzyme
Source: PLoS One. 2016 Apr 20;11(4):e0153360. doi: 10.1371/journal.pone.0153360 (PMC4838334; doi:10.1371/journal.pone.0153360)
Supplement: S1 Table — Digested peptide concentration was calculated according to the fluorescence intensity of qf-Aβ(12–16)AAC obtained from the reaction of NEP or ACE at 37°C overnight. (DOC) [file pone.0153360.s001.doc]

**Supporting Information**

**Design of peptide substrate for sensitively and specifically detecting two Aβ-degrading enzymes: neprilysin and angiotensin-converting enzyme**

Po-Ting Chen^1,2^, Chao-Long Chen^3^, Lilian Tsai-Wei Lin^3^, Chun-Hsien Lo^3^, Chaur-Jong Hu^4^, Rita P.-Y. Chen^1,2^,*, and Steven *S.-S.* Wang^3^,*

^1^Institute of Biochemical Sciences, National Taiwan University, Taipei 10617, Taiwan

^2^Institute of Biological Chemistry, Academia Sinica, Taipei 11529, Taiwan

^3^Department of Chemical Engineering, National Taiwan University, Taipei 10617, Taiwan

^4^Department of Neurology, Shuang-Ho Hospital, Taipei Medical University, Taipei 110, Taiwan

**S1 Table. Kinetic parameters for the hydrolysis of qf-Aβ(12-16)AAC by NEP and ACE. Digested peptide concentration was calculated according to the fluorescence intensity of qf-Aβ(12-16)AAC obtained from the reaction of NEP or ACE at 37 °C overnight.**

Enzyme Km (μM) kcat (min^-1^) kcat/ Km (min^-1^ μM^-1^)

NEP 12.9 ± 6.1 34.5 ± 11.6 2.7

ACE 8.7 ± 2.6 34.7 ± 5.3 4.0
