# Supplementary material for: Design of Peptide Substrate for Sensitively and Specifically Detecting Two Aβ-Degrading Enzymes: Neprilysin and Angiotensin-Converting Enzyme
Source: PLoS One. 2016 Apr 20;11(4):e0153360. doi: 10.1371/journal.pone.0153360 (PMC4838334; doi:10.1371/journal.pone.0153360)
Supplement: S2 Table — Dabcyl-VHHQKAAC-Alexa350 is the parent peptide. (DOC) [file pone.0153360.s002.doc]

**Supporting Information**

**Design of peptide substrate for sensitively and specifically detecting two Aβ-degrading enzymes: neprilysin and angiotensin-converting enzyme**

Po-Ting Chen^1,2^, Chao-Long Chen^3^, Lilian Tsai-Wei Lin^3^, Chun-Hsien Lo^3^, Chaur-Jong Hu^4^, Rita P.-Y. Chen^1,2^,*, and Steven *S.-S.* Wang^3^,*

^1^Institute of Biochemical Sciences, National Taiwan University, Taipei 10617, Taiwan

^2^Institute of Biological Chemistry, Academia Sinica, Taipei 11529, Taiwan

^3^Department of Chemical Engineering, National Taiwan University, Taipei 10617, Taiwan

^4^Department of Neurology, Shuang-Ho Hospital, Taipei Medical University, Taipei 110, Taiwan

**S2 Table.** Summary of the sequences, theoretical masses [M], and observed masses [M+H^+^] of the peptide fragments obtained from the MALDI spectrometric analysis shown in S3 Fig. Dabcyl-VHHQKAAC-Alexa350 is the parent peptide.

| Peptide sequence | Theoretical mass [M] | Observed mass [M+H^+^] |
| --- | --- | --- |
| Dabcyl-VHHQKAAC-Alexa350 | 1621 | - |
| AAC-Alexa350 | 740 | 741.187 |
| AC-Alexa350 | 669 | 670.130 |
