# Supplementary material for: Design of Peptide Substrate for Sensitively and Specifically Detecting Two Aβ-Degrading Enzymes: Neprilysin and Angiotensin-Converting Enzyme
Source: PLoS One. 2016 Apr 20;11(4):e0153360. doi: 10.1371/journal.pone.0153360 (PMC4838334; doi:10.1371/journal.pone.0153360)
Supplement: S2 Fig — 2 nM NEP or ACE was reacted with 1 to 15 μM of qf-Aβ(12–16)AAC in 50 mM Tris-HCl (pH 7.5), 25 mM NaCl, 5 βM ZnCl2 at 37. The fluorescence emission at 465 nm was recorded every minute on a Paradigm™ Detection Platform (Beckman Coulter, USA) with excitation at 360 nm. The initial digestion rates for different concentrations of substrate were obtained by non-linear fitting and plotted against the substrate concentrations. The curve fitting on the data of initial rate/velocity versus substrate concentration plot was conducted according to the standard Michaelis-Menten equation. (DOC) [file pone.0153360.s004.doc]

**Supporting Information**

**Design of peptide substrate for sensitively and specifically detecting two Aβ-degrading enzymes: neprilysin and angiotensin-converting enzyme**

Po-Ting Chen^1,2^, Chao-Long Chen^3^, Lilian Tsai-Wei Lin^3^, Chun-Hsien Lo^3^, Chaur-Jong Hu^4^, Rita P.-Y. Chen^1,2^,*, and Steven *S.-S.* Wang^3^,*

^1^Institute of Biochemical Sciences, National Taiwan University, Taipei 10617, Taiwan

^2^Institute of Biological Chemistry, Academia Sinica, Taipei 11529, Taiwan

^3^Department of Chemical Engineering, National Taiwan University, Taipei 10617, Taiwan

^4^Department of Neurology, Shuang-Ho Hospital, Taipei Medical University, Taipei 110, Taiwan

**S2 Fig.** Kinetics of qf-Aβ(12-16)AAC digestion by NEP and ACE. 2 nM NEP or ACE was reacted with 1 to 15 μM of qf-Aβ(12-16)AAC in 50 mM Tris-HCl (pH 7.5), 25 mM NaCl, 5 μM ZnCl_2_ at 37 °C. The fluorescence emission at 465 nm was recorded every minute on a Paradigm^TM^ Detection Platform (Beckman Coulter, USA) with excitation at 360 nm. The initial digestion rates for different concentrations of substrate were obtained by non-linear fitting and plotted against the substrate concentrations. The curve fitting on the data of initial rate/velocity versus substrate concentration plot was conducted according to the standard Michaelis-Menten equation.
