# Supplementary material for: Design of Peptide Substrate for Sensitively and Specifically Detecting Two Aβ-Degrading Enzymes: Neprilysin and Angiotensin-Converting Enzyme
Source: PLoS One. 2016 Apr 20;11(4):e0153360. doi: 10.1371/journal.pone.0153360 (PMC4838334; doi:10.1371/journal.pone.0153360)
Supplement: S3 Fig — SH-SY5Y cells were seeded in 100-mm dishes (10 mL; cell density of 7×10^5^ cells/mL), then, after 1 day, the medium was replaced with the fresh DMEM/F12 medium supplemented with 10% FBS and 10 μM of samatostatin (Sigma, USA) or vehicle. After incubation for 24 hr, cells were first detached from the plate with PBS and then centrifuged at 200 g at 4°C for 5 min. For the detection of IDE level in the differentiated cells, 5×10^5^ cells/mL in DMEM/F12 medium supplemented with 1% FBS was used. On the second day of incubation, the medium was replaced by the above-mentioned medium containing 10 μM retinoic acid (RA) and the cell culture was further incubated for 5 days (the medium should be changed every two days). The membrane fractions were extracted from the cell pellets using the Mem-PER Plus Membrane Protein Extraction Kit (Thermo Scientific, USA). After quantifying the protein content of the membrane fractions using a BCA kit (Thermo Scientific, USA), 50 μg of protein were first taken and resolved on an 8% Bis-Tris gels by SDS-PAGE and then transferred to a nitrocellulose membrane (PerkinElmer, USA). Next, the membrane was blocked with 5% non-fat dry milk (Fonterra, New Zealand) in the blocking buffer (Tris-buffered saline (TBS) containing 0.1% Tween 20) for 1 hr at 4°C, and incubated overnight at 4°C with mouse monoclonal anti-IDE antibody (Covance, USA) and mouse monoclonal anti-glyceraldehyde 3-phosphate dehydrogenase (GAPDH) antibody (Proteintech, USA) (1:1000 in the blocking buffer). After 3 washes with TBS, the membranes were incubated for 1 hr at 4°C with horseradish peroxidase-conjugated anti-mouse IgG antibodies (1:1000 in the blocking buffer, R&D Systems, USA). A slight increase in IDE amount was detected after somatostatin treatment. (DOC) [file pone.0153360.s005.doc]

**Supporting Information**

**Design of peptide substrate for sensitively and specifically detecting two Aβ-degrading enzymes: neprilysin and angiotensin-converting enzyme**

Po-Ting Chen^1,2^, Chao-Long Chen^3^, Lilian Tsai-Wei Lin^3^, Chun-Hsien Lo^3^, Chaur-Jong Hu^4^, Rita P.-Y. Chen^1,2^,*, and Steven *S.-S.* Wang^3^,*

^1^Institute of Biochemical Sciences, National Taiwan University, Taipei 10617, Taiwan

^2^Institute of Biological Chemistry, Academia Sinica, Taipei 11529, Taiwan

^3^Department of Chemical Engineering, National Taiwan University, Taipei 10617, Taiwan

^4^Department of Neurology, Shuang-Ho Hospital, Taipei Medical University, Taipei 110, Taiwan


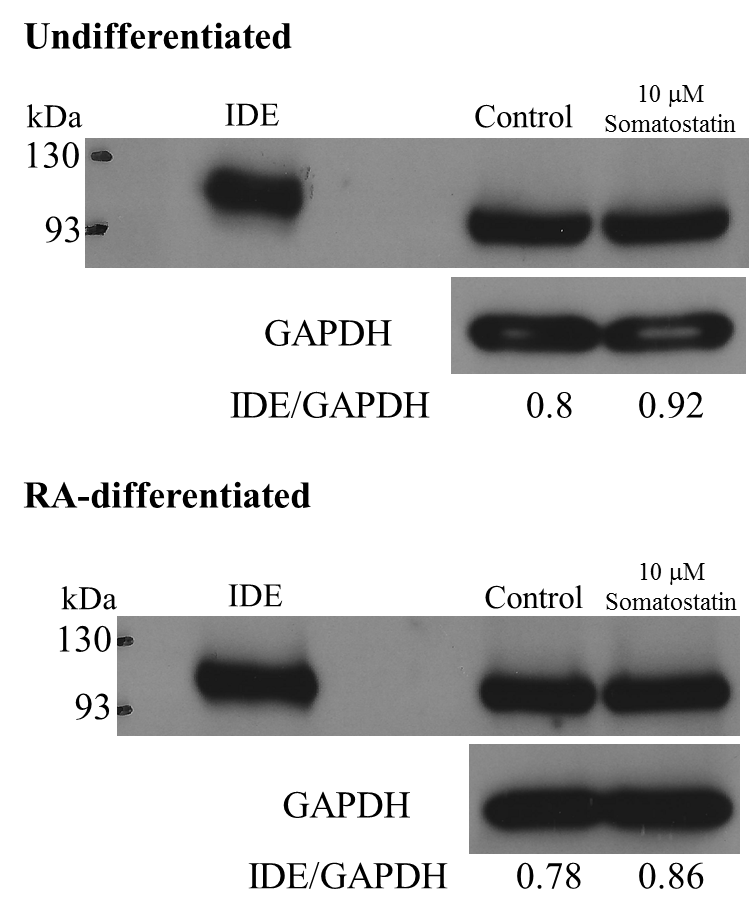


**S3 Fig.** Effect of somatostatin on IDE level by western blotting. SH-SY5Y cells were seeded in 100-mm dishes (10 mL; cell density of 7🞨10^5^ cells/mL), then, after 1 day, the medium was replaced with the fresh DMEM/F12 medium supplemented with 10% FBS and 10 μM of samatostatin (Sigma, USA) or vehicle. After incubation for 24 hr, cells were first detached from the plate with PBS and then centrifuged at 200 g at 4 ºC for 5 min. For the detection of IDE level in the differentiated cells, 5🞨10^5^ cells/mL in DMEM/F12 medium supplemented with 1% FBS was used. On the second day of incubation, the medium was replaced by the above-mentioned medium containing 10 μM retinoic acid (RA) and the cell culture was further incubated for 5 days (the medium should be changed every two days). The membrane fractions were extracted from the cell pellets using the Mem-PER Plus Membrane Protein Extraction Kit (Thermo Scientific, USA). After quantifying the protein content of the membrane fractions using a BCA kit (Thermo Scientific, USA), 50 μg of protein were first taken and resolved on an 8 % Bis-Tris gels by SDS-PAGE and then transferred to a nitrocellulose membrane (PerkinElmer, USA). Next, the membrane was blocked with 5 % non-fat dry milk (Fonterra, New Zealand) in the blocking buffer (Tris-buffered saline (TBS) containing 0.1 % Tween 20) for 1 hr at 4 ºC, and incubated overnight at 4 ºC with mouse monoclonal anti-IDE antibody (Covance, USA) and mouse monoclonal anti-glyceraldehyde 3-phosphate dehydrogenase (GAPDH) antibody (Proteintech, USA) (1:1000 in the blocking buffer). After 3 washes with TBS, the membranes were incubated for 1 hr at 4 ºC with horseradish peroxidase-conjugated anti-mouse IgG antibodies (1:1000 in the blocking buffer, R&D Systems, USA). A slight increase in IDE amount was detected after somatostatin treatment.
