# Supplementary material for: Design of Peptide Substrate for Sensitively and Specifically Detecting Two Aβ-Degrading Enzymes: Neprilysin and Angiotensin-Converting Enzyme
Source: PLoS One. 2016 Apr 20;11(4):e0153360. doi: 10.1371/journal.pone.0153360 (PMC4838334; doi:10.1371/journal.pone.0153360)
Supplement: S1 File — 1 mL 10 μM qf-Aβ(12–16)AAC in 50 mM Tris-HCl (pH 7.5), 25 mM NaCl, 5 μM ZnCl2 was reacted with 250 ng NEP or ACE at 37°C for 1 hr. The reaction product was purified by HPLC with C18 column. (A) HPLC chromatogram for NEP digestion. (C) HPLC chromatogram for ACE digestion. The elution gradient used was 0–50% Buffer B in 20 min. Buffer A: 5% acetonitrile/0.1% TFA in water; Buffer B: 0.1% TFA in acetonitrile. The products were detected by monitoring the absorbance at the wavelengths of 346 and 453 nm. After cleavage, the peptide fragment containing the Alexa-350 moiety exhibited a positive absorption peak at 346 nm but a negative absorption peak at 453 nm, which is due to the emitted fluorescence from Alexa-350. However, the peptide fragment containing the Dabcyl moiety showed absorption at 453 nm. The peak eluted at 12 min (with absorption at 346 nm) was collected and further identified/characterized by MALDI-TOF mass spectroscopy. (B) MALDI-TOF mass spectrum of the sample pointed with an arrow in the chromatogram of NEP digestion product. (D) MALDI-TOF mass spectrum of the sample pointed with an arrow in the chromatogram of ACE digestion product. The observed mass [M+H+] in (B) was determined to be 741.187 Da, which corresponds to the mass of the peptide fragment AAC-Alexa-350 with a theoretical mass of 740 Da. This result indicated that the cutting site of NEP is located between the residues K and A in the sequence “VHHQKAAC”. The observed mass [M+H+] in (D) was determined to be 670.130 Da, which is ~71 Da lower than the value obtained in the case of NEP digestion. Since this amount of mass reduction is equal to the mass of Ala residue, we believe the peak belongs to the peptide fragment AC-Alexa-350. This finding indicated that the cutting site of ACE is located between two Ala residues in the sequence “VHHQKAAC”. (DOC) [file pone.0153360.s006.doc]

**Supporting Information**

**Design of peptide substrate for sensitively and specifically detecting two Aβ-degrading enzymes: neprilysin and angiotensin-converting enzyme**

Po-Ting Chen^1,2^, Chao-Long Chen^3^, Lilian Tsai-Wei Lin^3^, Chun-Hsien Lo^3^, Chaur-Jong Hu^4^, Rita P.-Y. Chen^1,2^,*, and Steven *S.-S.* Wang^3^,*

^1^Institute of Biochemical Sciences, National Taiwan University, Taipei 10617, Taiwan

^2^Institute of Biological Chemistry, Academia Sinica, Taipei 11529, Taiwan

^3^Department of Chemical Engineering, National Taiwan University, Taipei 10617, Taiwan

^4^Department of Neurology, Shuang-Ho Hospital, Taipei Medical University, Taipei 110, Taiwan


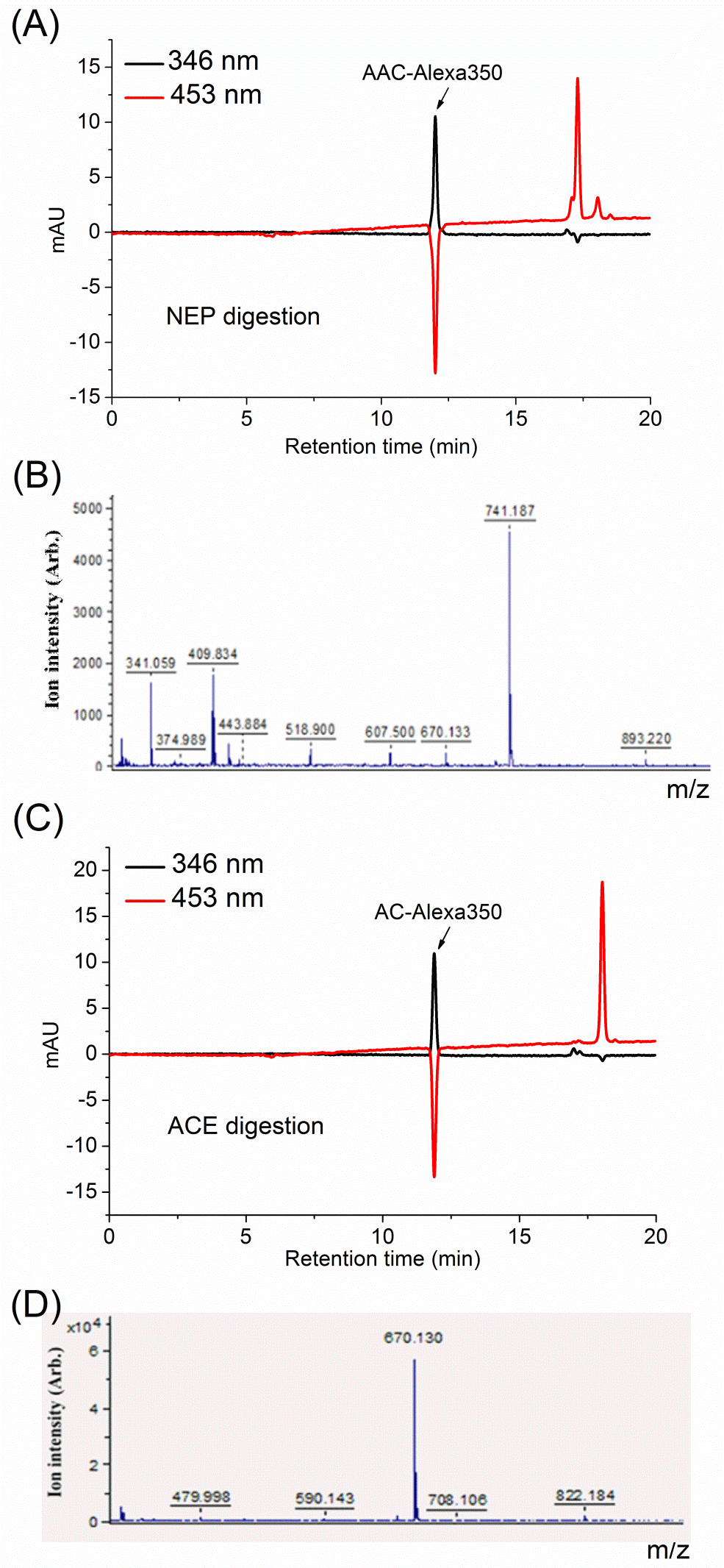


**S1 File.** Analysis of the cutting site of NEP or ACE on qf-Aβ(12-16)AAC. 1 mL 10 μM qf-Aβ(12-16)AAC in 50 mM Tris-HCl (pH 7.5), 25 mM NaCl, 5 μM ZnCl_2_ was reacted with 250 ng NEP or ACE at 37 °C for 1 hr. The reaction product was purified by HPLC with C18 column. (A) HPLC chromatogram for NEP digestion. (C) HPLC chromatogram for ACE digestion. The elution gradient used was 0-50 % Buffer B in 20 min. Buffer A: 5 % acetonitrile/0.1 % TFA in water; Buffer B: 0.1 % TFA in acetonitrile. The products were detected by monitoring the absorbance at the wavelengths of 346 and 453 nm. After cleavage, the peptide fragment containing the Alexa-350 moiety exhibited a positive absorption peak at 346 nm but a negative absorption peak at 453 nm, which is due to the emitted fluorescence from Alexa-350. However, the peptide fragment containing the Dabcyl moiety showed absorption at 453 nm. The peak eluted at 12 min (with absorption at 346 nm) was collected and further identified/characterized by MALDI-TOF mass spectroscopy. (B) MALDI-TOF mass spectrum of the sample pointed with an arrow in the chromatogram of NEP digestion product. (D) MALDI-TOF mass spectrum of the sample pointed with an arrow in the chromatogram of ACE digestion product. The observed mass [M+H^+^] in (B) was determined to be 741.187 Da, which corresponds to the mass of the peptide fragment AAC-Alexa-350 with a theoretical mass of 740 Da. This result indicated that the cutting site of NEP is located between the residues K and A in the sequence “VHHQKAAC”. The observed mass [M+H^+^] in (D) was determined to be 670.130 Da, which is ~71 Da lower than the value obtained in the case of NEP digestion. Since this amount of mass reduction is equal to the mass of Ala residue, we believe the peak belongs to the peptide fragment AC-Alexa-350. This finding indicated that the cutting site of ACE is located between two Ala residues in the sequence “VHHQKAAC”.
